# Soluble THSD7A Is an N-Glycoprotein That Promotes Endothelial Cell Migration and Tube Formation in Angiogenesis

**DOI:** 10.1371/journal.pone.0029000

**Published:** 2011-12-14

**Authors:** Meng-Wei Kuo, Chian-Huei Wang, Hsiao-Chun Wu, Shing-Jyh Chang, Yung-Jen Chuang

**Affiliations:** 1 Department of Medical Science and Institute of Bioinformatics and Structural Biology, National Tsing Hua University, Hsinchu, Taiwan, Republic of China; 2 Department of Obstetrics and Gynecology, Hsinchu Mackay Memorial Hospital, Hsinchu, Taiwan, Republic of China; University of Birmingham, United Kingdom

## Abstract

**Background:**

Thrombospondin type I domain containing 7A (THSD7A) is a novel neural protein that is known to affect endothelial migration and vascular patterning during development. To further understand the role of THSD7A in angiogenesis, we investigated the post-translational modification scheme of THS7DA and to reveal the underlying mechanisms by which this protein regulates blood vessel growth.

**Methodology/Principal Findings:**

Full-length *THSD7A* was overexpressed in human embryonic kidney 293T (HEK293T) cells and was found to be membrane associated and N-glycosylated. The soluble form of THSD7A, which is released into the cultured medium, was harvested for further angiogenic assays. We found that soluble THSD7A promotes human umbilical vein endothelial cell (HUVEC) migration and tube formation. HUVEC sprouts and zebrafish subintestinal vessel (SIV) angiogenic assays further revealed that soluble THSD7A increases the number of branching points of new vessels. Interestingly, we found that soluble THSD7A increased the formation of filopodia in HUVEC. The distribution patterns of vinculin and phosphorylated focal adhesion kinase (FAK) were also affected, which implies a role for THSD7A in focal adhesion assembly. Moreover, soluble THSD7A increased FAK phosphorylation in HUVEC, suggesting that THSD7A is involved in regulating cytoskeleton reorganization.

**Conclusions/Significance:**

Taken together, our results indicate that THSD7A is a membrane-associated N-glycoprotein with a soluble form. Soluble THSD7A promotes endothelial cell migration during angiogenesis via a FAK-dependent mechanism and thus may be a novel neuroangiogenic factor.

## Introduction

Angiogenesis is the process of new vascular growth from pre-existing blood vessels. It has been shown to be a critical process in embryonic development and growth as well as in wound healing and tumor progression. The current consensus on the molecular mechanism of angiogenesis suggests that this vital process occurs in several stages, which are all tightly regulated and balanced by both pro- and anti-angiogenic factors. In the presence of pro-angiogenic stimulation, the endothelium is induced to degrade the adjacent extracellular matrix (ECM). This degradation allows activated endothelial cells to migrate out of the original blood vessels. These endothelial cells then proliferate and arrange into sprouts, which extend toward the source of angiogenic stimulation. During development, guidance molecules and anti-angiogenic factors act together to ensure that such angiogenic sprouting follows a prescribed path to connect with neighboring vessels. These sprouts will eventually loop, stabilize and mature into functional vessels with lumen to allow blood circulation [1]–[3].

During angiogenic sprouting and branch formation, endothelial cells react to different signals and commit to distinct cellular fates. For example, tip cells sense and respond to guidance molecules with filopodia [4]–[8]. During cell migration, the filopodia at the tip cell leading edge can sense the microenvironment and drive directed cell migration. Previous studies have shown that integrins located at filopodia can probe the surrounding matrix and create ‘sticky fingers’ along the leading edge. These structures, in turn, promote the assembly of focal adhesion complexes to stabilize protrusions and promote migration [9]–[10]. FAK, an important member of focal adhesion complexes, is activated when a cell is stimulated by clustered integrins or other growth factors. FAK can recruit other focal adhesion components, such as vinculin and paxillin, and establish focal adhesions [11]–[14].

In a previous study, we identified THSD7A as a novel endothelial protein and found that it is preferentially expressed in the placental vasculature. We demonstrated that carboxyl-terminal fragments of THSD7A co-localized with aVb3 integrin and paxillin; in addition, overexpression and knockdown of THSD7A showed that THSD7A regulates cell mobility and tube formation in HUVEC [15]. In the other hand, zebrafish *thsd7a* transcripts are detected along the ventral edge of the neural tube in developing zebrafish, and THSD7A is required for the angiogenesis of intersegmental vessels (ISVs) [16]. In this study, we demonstrate that full-length THSD7A is expressed in HUVEC and SH-SY5Y neuroblastoma cells, and both can release a 210 kDa soluble form of the protein. We also investigate the post-translational modification of THSD7A and the protein's functional mechanism. We discovered that a soluble form of THSD7A is released into the extracellular environment. By various *in vitro* and *in vivo* angiogenic assays, we demonstrate that soluble THSD7A promotes endothelial filopodia formation and focal adhesion assembly and induces FAK-dependent signaling during angiogenesis. Taken together with the results of our previous study, our findings indicate that soluble THSD7A is a potent neuroangiogenic factor.

## Materials and Methods

A detailed methods description is included in the Data S1. Soluble THSD7A was collected from *THSD7A*-transfected HEK293T cell culture medium. Cell culture medium from empty vector-transfected HEK293T cells served as control. Experiments were performed in primary HUVEC. Migration rate was analyzed by transwell migration assays using polycarbonate filters (8 µm pore size). Two-dimensional tube formation was performed on Matrigel. Three-dimensional tube formation was performed in type I collagen matrix and served as a measure of *in vitro* sprouting ability. Analysis of branching of the zebrafish subintestinal vessel (SIV) served as an *in vivo* angiogenesis assay. HUVEC adhesion, filopodia formation, and vinculin and FAK pY397 distribution assays were performed on collagen-coated coverslips. All of the zebrafish use protocols in this research were reviewed and approved by the Institutional Animal Care and Use Committee of National Tsing Hua University (IRB Approval NO. 09507).

## Results

### Native THSD7A has a soluble form and is expressed by HUVEC and SH-SY5Y cells

**Figure 1:**
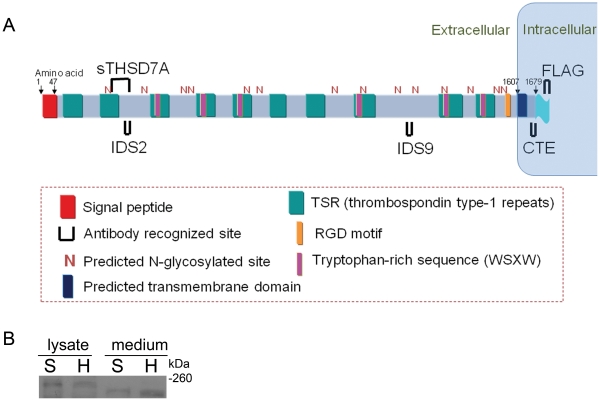
Domain organization prediction of full-length THSD7A and epitope targeting sites. **A**. Based on domain prediction analysis, THSD7A contains an extracellular signal peptide at the amino terminus, ten thrombospondin type 1 repeats, six WSXW motifs, one RGD motif, one predicted transmembrane domain and fourteen predicted N-glycosylated sites. A FLAG-tag was fused to the carboxyl terminus of the full-length *THSD7A*. The recognition sites of THSD7A-specific antibodies (sTHSD7A, IDS2, IDS9 and CTE) and the FLAG-tag are marked as **∪**. **B**. Cell lysates and cultured medium of HUVEC (H) and SH-SY5Y (S) cells were analyzed by Western blot using anti-sTHSD7A antibody.

To investigate the role of THSD7A in angiogenesis, we purified THSD7A-specific custom antibodies (anti-IDS2, anti-IDS9 and anti-CTE) for further study. Commercial antibodies (anti-sTHSD7A and anti-FLAG-tag) were also used to confirm the results (Figure 1A). THSD7A was expressed in HUVEC lysates based on anti-CTE antibody staining [15]. In addition, analysis of the human *THSD7A* expression profile (UniGene number Hs. 120855) in the NCBI dbEST database revealed that *THSD7A* is also highly expressed in tissues such as the brain and nerves. Therefore, we compared the THSD7A protein expression patterns in cell lysates and cultured medium from SH-SY5Y cells (a human-derived neuroblastoma cell line) to those of HUVEC by Western blot. As shown in Figure 1B, a 260 kDa protein was recognized by the anti-sTHSD7A antibody in both HUVEC and SH-SY5Y cell lysates. However, the anti-sTHSD7A antibody instead recognized a ∼210 kDa protein in the cultured medium (Figure 1B). These data demonstrate that native THSD7A has a soluble form with a reduced molecular weight of ∼210 kDa.

### Soluble THSD7A is released from membrane-associated N-glycosylated THSD7A

**Figure 2:**
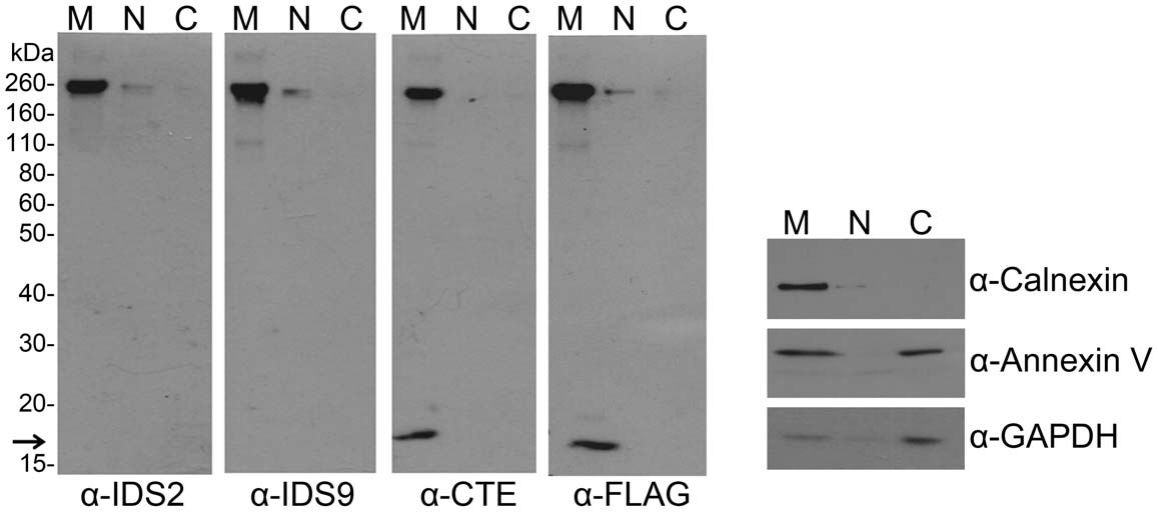
THSD7A is a membrane-associated protein. *THSD7A*-transfected HEK293T cells were homogenized and the cytosolic (C), nuclear (N) and membrane (M) fractions were prepared as described in the materials and methods. Each fraction was subjected to Western blot with anti-THSD7A (IDS2, IDS9, CTE), anti-FLAG-tag, anti-calnexin (plasma membrane marker), anti-annexin V (cytoplasm and plasma membrane marker) and anti-GAPDH (cytoplasm marker) antibodies. Arrow indicates the carboxyl-terminal fragment of THSD7A.

After discovering that native THSD7A has a soluble form, we decided to investigate its post-translational modifications and trafficking. First, we examined the subcellular distribution of THSD7A protein using cell fractionation and Western blotting of HEK293T cells transfected with recombinant FLAG-tagged full-length *THSD7A* (Figure 2). Our data showed that all of the THSD7A-specific and anti-FLAG-tag antibodies recognized the same ∼260 kDa band in the membrane fraction, suggesting that full-length THSD7A is membrane associated (M lanes in Figure 2). In these experiments, calnexin served as a plasma membrane marker and GAPDH served as a cytoplasmic marker. Annexin V served as both a cytoplasmic and plasma membrane marker (Figure 2, right panel).

**Figure 3:**
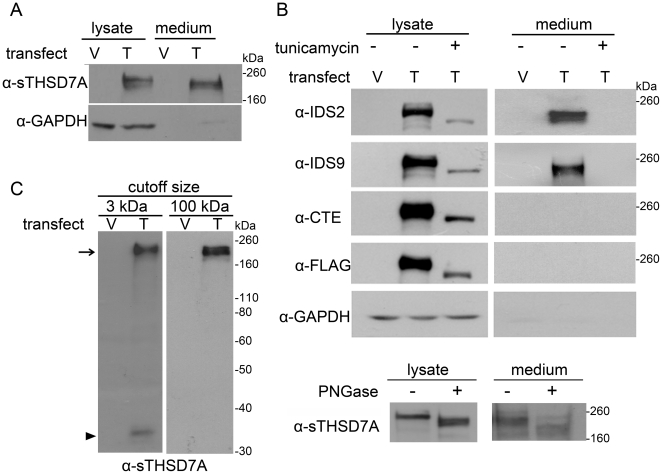
THSD7A is an N-glycoprotein and can release the soluble form into the extracellular environment. **A**. Empty vector (V)- and *THSD7A* (T)-transfected HEK293T cells were analyzed by Western blot. THSD7A was detected in both cell lysates and cultured medium. GAPDH served as a cytoplasmic marker. **B**. Transfected HEK293T cells were incubated in the presence (+) or absence (−) of tunicamycin. After 48 hours, cell lysates and cultured medium were subjected to Western blot and stained with THSD7A-specific and anti-FLAG-tag antibodies (upper panel). The cell lysates and cultured medium of *THSD7A*-transfected HEH293T cells were treated with (+) or without (−) PNGase and subjected to Western blot (lower panel). **C**. Cultured medium from transfected HEK293T cells was concentrated by 3 kDa or 100 kDa cutoff Amicon concentrators and analyzed by Western blot. After concentration by a 100 kDa cutoff Amicon concentrator, the 210 kDa soluble THSD7A (arrow) was retained in the concentrates and the 33 kDa amino-terminal fragment of THSD7A (arrowhead) was filtered out.

Interestingly, the anti-CTE and anti-FLAG-tag antibodies also detected a ∼17 kDa fragment (M lanes in Figure 2, arrow). As described in Figure 1A, these antibodies recognized sites near the carboxyl-terminal region of THSD7A. The results showed that this ∼17 kDa fragment was also enriched in the membrane fraction. These data suggest that full-length THSD7A is a membrane-associated protein with a molecular weight of 260 kDa, and it might be cleaved to release a soluble form while leaving a 17 kDa carboxyl-terminal fragment in the cell membrane. Next, we collected and analyzed by Western blot the lysates and cultured medium from *THSD7A*-transfected HEK293T cells. The soluble THSD7A presented in the cultured medium had a size of ∼210 kDa, which is smaller than the full-length THSD7A (Figure 3A). This result is consistent with the result shown in Figure 1B.

To identify post-translational modifications of THSD7A, we conducted further analyses of *THSD7A*-transfected HEK293T cell lysates and cultured medium. *In silico* ExPASy analysis predicted that THSD7A would have a net molecular weight of ∼186 kDa without any post-translational modification. This weight is significantly lower than the ∼260 kDa band we observed in this study. We hypothesized that the difference between the predicted and apparent molecular weight of THSD7A is caused by extensive N-glycosylation. To test this hypothesis, we treated *THSD7A-*transfected HEK293T cells with tunicamycin, which is an inhibitor of GlcNAc phosphotransferase, the enzyme responsible for the first step of the N-glycosylation pathway (Figure 3B, upper panel). In the absence of tunicamycin, we could again detect the ∼260 kDa form of THSD7A in cell lysates and the ∼210 kDa form of soluble THSD7A in cultured medium. However, with the addition of tunicamycin, the observed molecular weight of THSD7A in the cell lysate was reduced to ∼190 kDa, and no soluble THSD7A could be detected in the cultured medium. N-glycosylation of THSD7A was also demonstrated by PNGase treatment (Figure 3B, lower panel). Furthermore, an additional ∼33 kDa amino-terminal fragment was detectable using the anti-sTHSD7A antibody in the cultured medium of transfected cells that did not receive tunicamycin treatment (Figure 3C, left panel, arrow head). This 33 kDa fragment was retained by a 3 kDa cutoff Amicon concentrator, but not by a 100 kDa cutoff Amicon concentrator (Figure 3C, right panel). Taken together, these results indicate that soluble THSD7A is likely to be cleaved at two sites: between the CTE and IDS9 epitopes and within the recognition site of the anti-sTHSD7A antibody (see Figure 1A for the epitope map).

Because THSD7A has been shown to play a role in angiogenesis [16], we were interested in learning whether the soluble 210 kDa form of THSD7A is responsible for regulating endothelial cells. We therefore concentrated the *THSD7A*-transfected HEK293T cell cultured medium using an Amicon concentrator (100 kDa cutoff size) to retain the ∼210 kDa soluble THSD7A (Figure 3C, right panel, arrow) for subsequent functional assays.

### Soluble THSD7A accelerates endothelial cell migration, two-dimensional tube formation and three-dimensional sprout formation

Based on the results of our previous study [15] and those shown in Figure 1B, we concluded that HUVECs produce soluble THSD7A. To determine whether this endogenous soluble THSD7A affects angiogenesis, we performed an enzyme-linked immunosorbent assay to detect soluble THSD7A in the cultured medium of HUVEC. After 48 hrs of culture, the concentration of HUVEC-released endogenous soluble THSD7A in the cultured medium was less than 0.1 ng/ml and below detectable limits. Similarly, in the concentrated cell culture medium of control vector-transfected HEK293T cells, the concentration of soluble THSD7A was also less than 0.1 ng/ml. In contrast, the concentration of soluble THSD7A in the cell cultured medium of *THSD7A-*transfected HEK293T cells was ∼75.5 ng/ml (∼360 nM); thus, endogenous soluble THSD7A should not affect our angiogenic assays.

**Figure 4:**
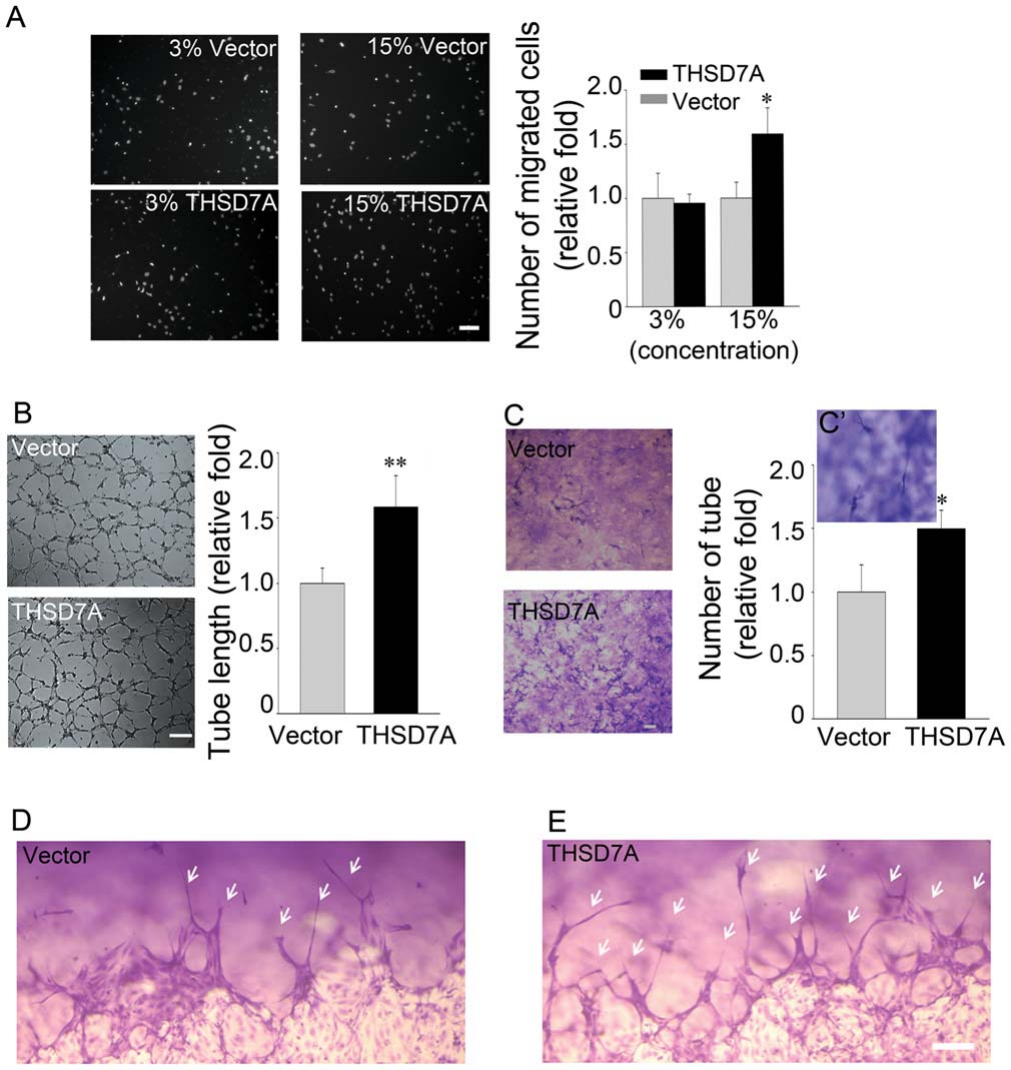
Soluble THSD7A accelerates endothelial cell migration, tube formation and sprouting. **A**. The motility of HUVEC was evaluated by transwell migration assay in M200 medium. HUVEC were loaded in the upper insert and soluble THSD7A or control medium were added to a 3% or 15% final concentration into the lower chamber. **B**. Two-dimensional tube formation was performed in the presence or absence of soluble THSD7A on Matrigel. **C**. Three-dimensional tube formation was performed in the presence or absence of soluble THSD7A on type I collagen as an assay for sprouting. **C′**. Enlarged image of tubes. Bar represents 10 µm. Each experiment was repeated at least three independent times. *P<0.05 vs. vector. **D–E**. Side views of the sprouting assay. Arrows indicate tubes. Bar represents 100 µm.

We used a transwell assay to determine whether soluble THSD7A affects endothelial cell migration *in vitro*. As shown in Figure 4A, HUVEC motility was not significantly different between cells treated with 3% soluble THSD7A (∼10.8 nM soluble THSD7A final concentration) and the vector-only control. However, when 15% soluble THSD7A (∼53.9 nM soluble THSD7A final concentration) was used, endothelial cell migration was increased by 59% compared to the vector-only control (Figure 4A).

We next examined the effect of soluble THSD7A on HUVEC tube formation. We found that when 25% soluble THSD7A (∼89.8 nM soluble THSD7A final concentration) was added into the medium, the formation of capillary-like networks on Matrigel was significantly enhanced. Compared to the vector-only control, the mean tube length of the HUVEC tubular network increased by 57% in the presence of soluble THSD7A (Figure 4B). To further confirm the angiogenic effect of soluble THSD7A, we performed a three-dimensional tube formation assay, which examines sprouting during angiogenesis, on HUVEC in a collagen matrix [17]–[18]. Our data show that, in the presence of soluble THSD7A, the capillary tube density was 50% higher than that of the vector-only control group (Figure 4C, top view of the 3D matrices). Side views of the three-dimensional endothelial sprouting assays clearly revealed that the number and length of endothelial sprouts increased in the presence of soluble THSD7A (Figure 4D–E). Taken together, these data support our hypothesis that soluble THSD7A could act as an angiogenic factor to promote endothelial cell migration, tube formation and sprouting.

### Soluble THSD7A induces angiogenic branching and abnormal vessel patterning in zebrafish embryos

**Figure 5:**
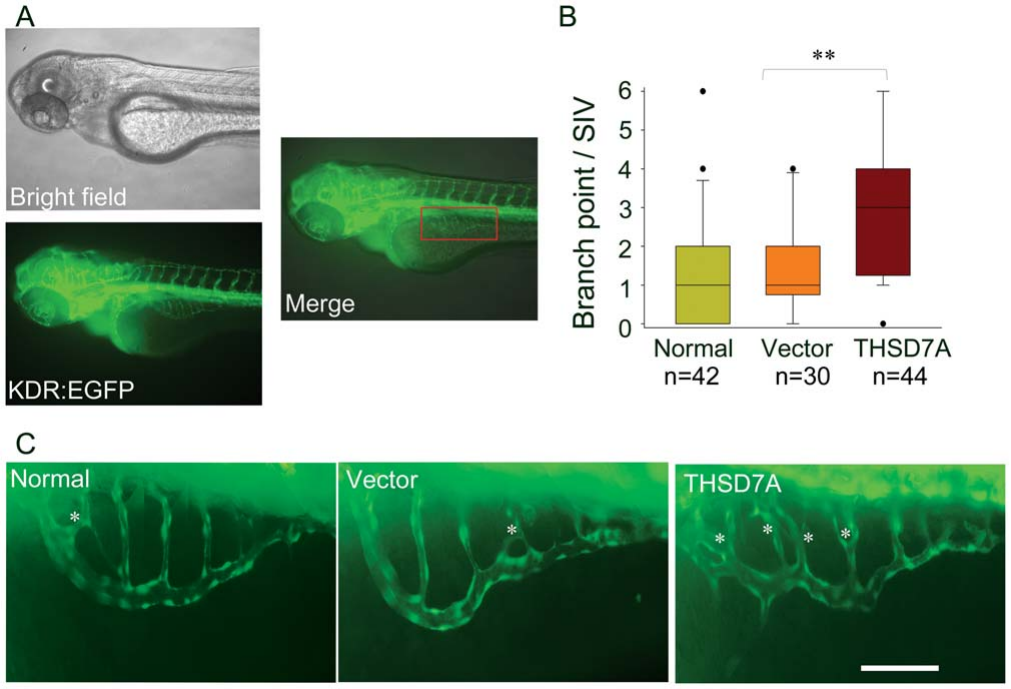
Soluble THSD7A increases the branching of SIVs and leads to an irregular vessel network in zebrafish. **A**. Images of 74 hpf Tg(kdr:EGFP) zebrafish. The red rectangle indicates the region of the SIV network. **B**. The median of branch point per SIV network of 74 hpf zebrafish. ** *P*<0.01 vs. vector. **C**. Enlarged images of SIV networks. * indicates branch points. Bar represents 100 µm.

To test the angiogenic function of soluble THSD7A *in vivo*, transgenic zebrafish Tg(kdr:EGFP) embryos, which have fluorescent vasculature, served as a model system for angiogenesis, as previously described [19]–[21]. Soluble THSD7A (9.2 nl; ∼0.69 ng) or control medium was injected into the zebrafish embryo yolk at 50 hours post fertilization (hpf). After 24 hours, the extent of development of the SIV network (Figure 5A, SIV is outlined by a red rectangle) was imaged and analyzed. Embryos without injection (normal) or those injected with the vector-only medium served as controls (Figure 5C, left and middle panels). Compared to the control groups, soluble THSD7A significantly increased the median number of branching points per SIV network from 1 to 3 (Figure 5B; Figure 5C, right panel). In addition, excrescent angiogenic sprouts were observed in the soluble THSD7A-treated SIV network, resulting in abnormal SIV patterning (Figure 5C). These results are in agreement with our *in vitro* data and validate that soluble THSD7A can also promote angiogenesis *in vivo*.

### Soluble THSD7A promotes filopodia formation and focal adhesion assembly to enhance cell migration via a FAK-involved manner

**Figure 6:**
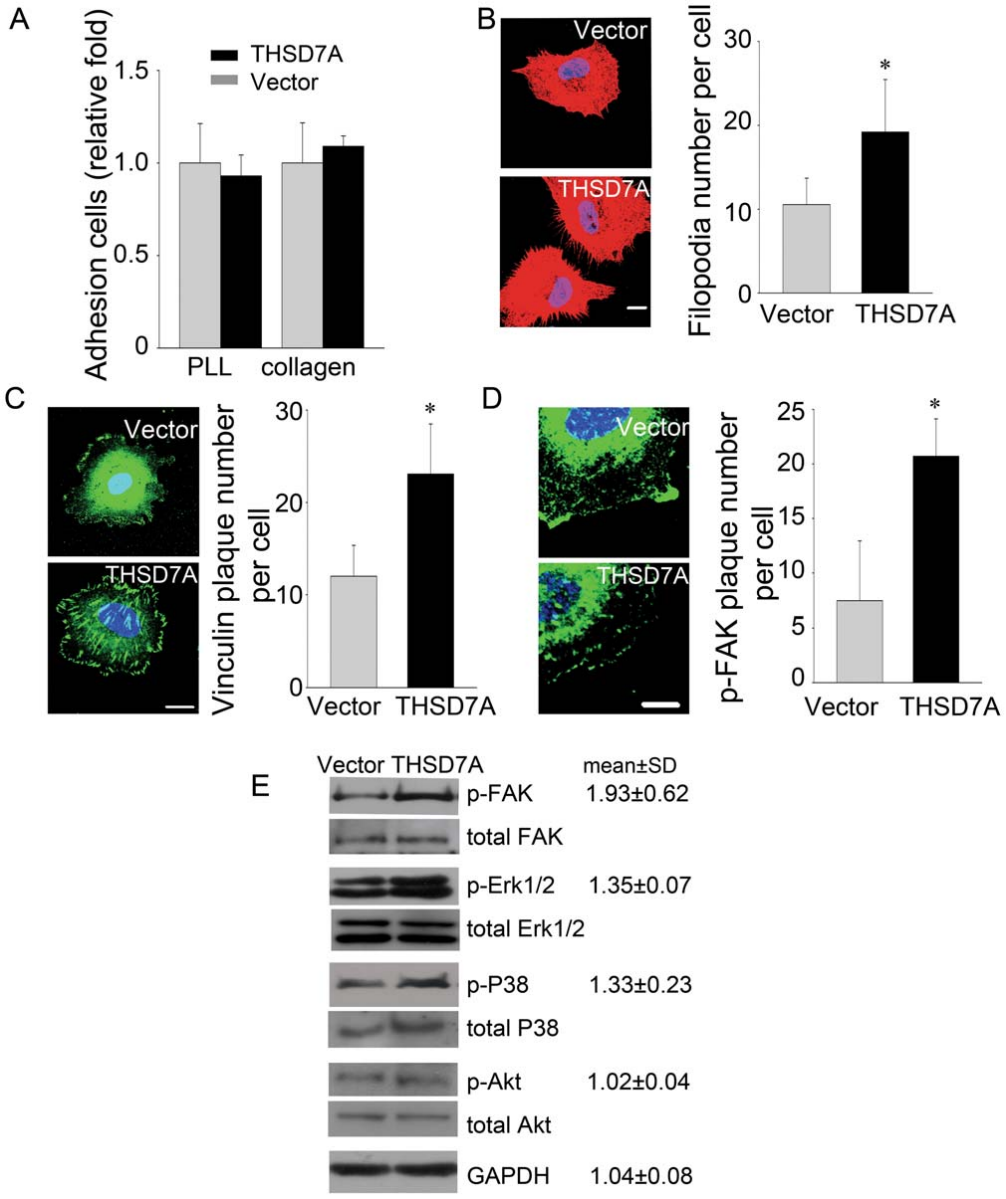
Soluble THSD7A promotes focal adhesion assembly to enhance cell migration via FAK-dependent manner. **A**. Adhesion assay performed with HUVEC in the presence and absence of soluble THSD7A on type I collagen- or PLL-coated coverslips. **B**. Immunostaining of actin filaments (red) and nuclei (blue) in HUVEC. The quantitative data show the average number of filopodia per cell. **C**. Immunostaining of vinculin (green) and nuclei (blue) in HUVEC. The quantitative data show the average number of peripheral vinculin plaques per cell. **D**. Immunostaining of p-FAK (green) and nuclei (blue) in HUVEC. The quantitative data show the average number of peripheral p-FAK plaques per cell. Bar represents 10 µm. *P<0.05 vs. vector. **E**, HUVEC lysates treated with soluble THSD7A or control medium were subjected to Western blot. Relative fold levels of p-FAK, p- Erk1/2, p-P38, p-Akt and GAPDH are represented as the mean ± SD. Total levels of FAK, Erk1/2, P38 and Akt served as loading controls. Each experiment was repeated at least three independent times.

Sprouting angiogenesis occurs in several well-characterized stages [4]–[5]. To determine which stage of angiogenesis is affected by soluble THSD7A, we performed a series of assays. First, by endothelial cell adhesion assay, we found no significant difference between the total numbers of adherent HUVEC in the presence of soluble THSD7A compared to the control group when cells were plated on type I collagen- or poly-L-lysine (PLL)-coated coverslips (Figure 6A). Thus, soluble THSD7A does not appear to affect endothelial cell adhesion. Because soluble THSD7A could promote endothelial cell migration, tube formation and sprouting (Figure 4,5), we hypothesized that soluble THSD7A might function in the initial steps of cell migration.

We next examined filopodia formation. We found that 20% soluble THSD7A (∼71.8 nM soluble THSD7A final concentration) significantly promoted microspike formation after HUVEC were seeded for 50 minutes, which were seen as slender cytoplasmic projections that extended beyond the leading edge of lamellipodia of HUVEC from 10 to 19 projections per cell (Figure 6B). In addition to filopodia formation, cell migration is also affected by focal adhesion assembly [10], [22]. The assembly, disassembly and turnover rates of focal adhesions are known to affect focal adhesion number and size. We used 20% soluble THSD7A to examine focal adhesion number by measuring the number of peripheral vinculin plaques; vinculin is a key adapter protein at the focal adhesion site and is known to stabilize filopodia [23]. After HUVEC were seeded for 50 minutes in the presence of soluble THSD7A, the number of well-distributed peripheral vinculin plaques (oval- or nail-shaped patterns around the attached cell) [23] increased by two-fold compared to the vector-only control (Figure 6C). We then investigated whether FAK, the phosphorylation of which is known to regulate focal adhesions during cell migration [22], [24], was affected by THSD7A. In the presence of soluble THSD7A, the number of well-distributed peripheral FAK pY397 plaques increased by three-fold (Figure 6D).

We then asked which cell migration signaling pathway is affected by soluble THSD7A. As expected, the phosphorylation level of FAK (pY397) in HUVEC increased by 93% when cells were stimulated with 20% soluble THSD7A for 15 minutes. Furthermore, the phosphorylation levels of Erk1/2 (pT202/R204) and P38 (pT180/R182) also increased by 35% and 33%, respectively. It is worth mentioning that the phosphorylation level of Akt (pS473) was not affected by soluble THSD7A. GAPDH was used as a loading control (Figure 6E). These results support the hypothesis that soluble THSD7A affects filopodia formation and focal adhesion assembly during cell migration in angiogenesis via a FAK-dependent mechanism.

### The specificity of soluble THSD7A-mediated angiogenic effects was demonstrated by blocking the activity with anti-sTHSD7A antibody

**Figure 7:**
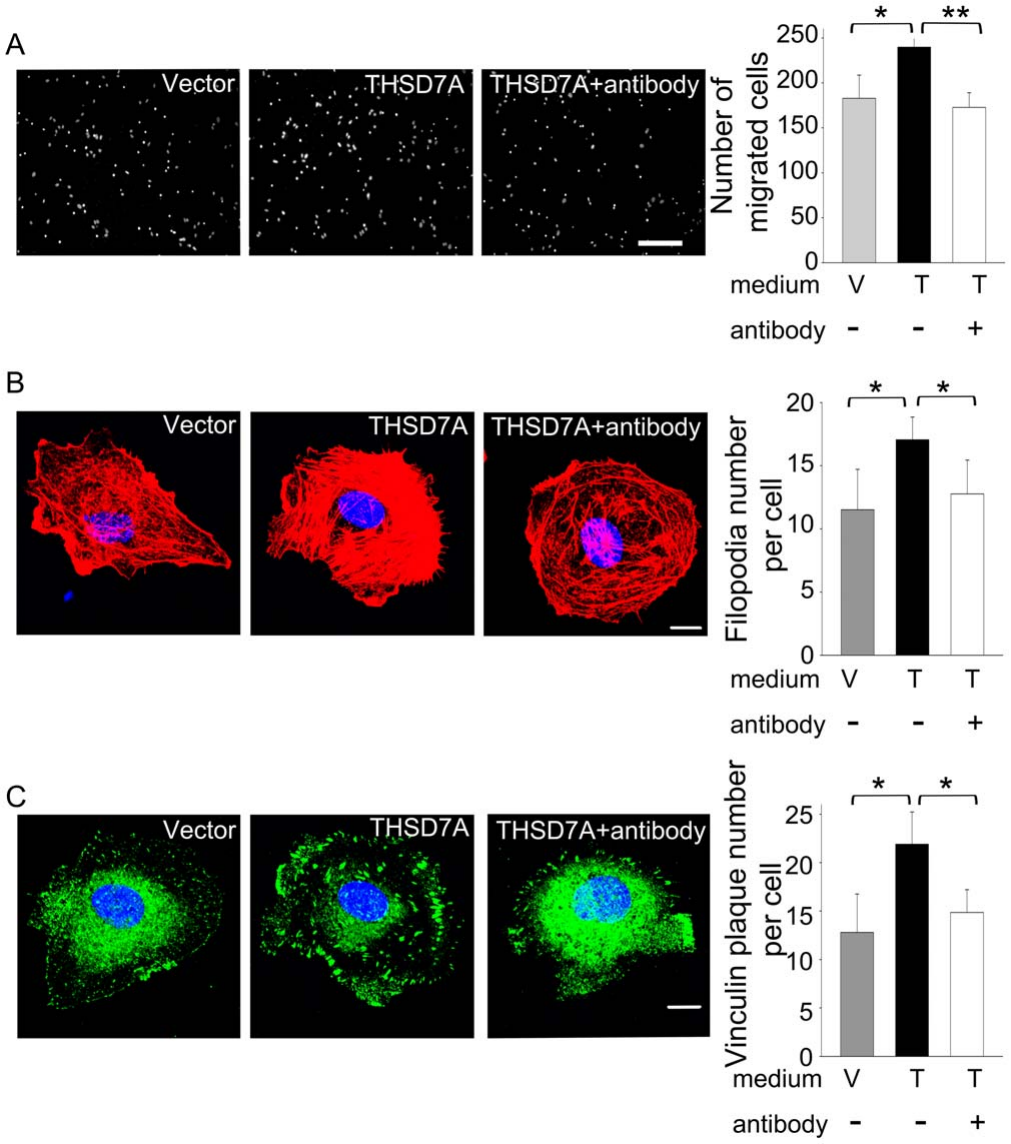
Soluble THSD7A-mediated angiogenic effects are blocked with anti-sTHSD7A antibody. **A.** The motility of HUVEC was evaluated by transwell migration assay. HUVEC were loaded in the upper insert. Control medium (V) or soluble THSD7A (T) in the presence (+) or absence (−) of anti-sTHSD7A antibody were added to the lower chamber. Bar represents 100 µm. **B.** Immunostaining of actin filaments (red) and nuclei (blue) in HUVEC treated with control medium (V) or soluble THSD7A (T) in the presence (+) or absence (−) of anti-sTHSD7A antibody. The quantitative data show the average number of filopodia per cell. **C.** Immunostaining of vinculin (green) and nuclei (blue) in HUVEC treated with control medium (V) or soluble THSD7A (T) in the presence (+) or absence (−) of anti-sTHSD7A antibody. The quantitative data show the average number of peripheral vinculin plaques per cell. Bar represents 10 µm. *P<0.05. **P<0.01. Each experiment was repeated at least three independent times.

To demonstrate the specificity of soluble THSD7A-mediated angiogenic effects, we used anti-sTHSD7A antibody to neutralize the activity of soluble THSD7A. First, we performed transwell assay to test whether the endothelial cell motility would be affected by the addition of anti-sTHSD7A antibody. As shown in Figure 7A, HUVEC motility in the antibody-added group was decreased by 39% as compared to the soluble THSD7A-treated group (which contained ∼71.8 nM soluble THSD7A). This data confirmed that soluble THSD7A promotes endothelial cell migration specifically. Furthermore, we examined the filopodia formation in the presence and absence of anti-sTHSD7A antibody. Compared with the soluble THSD7A-treated group, we found the addition of antibody decreased the number of filopodia from 17 to 12 per cell (Figure 7B). The number of well-distributed peripheral vinculin plaques in antibody-added group was also significantly decreased from 22 to 15 compared with the soluble THSD7A-treated group (Figure 7C). Taken these data together, soluble THSD7A indeed promote angiogenesis with significant specificity.

## Discussion

**Figure 8:**
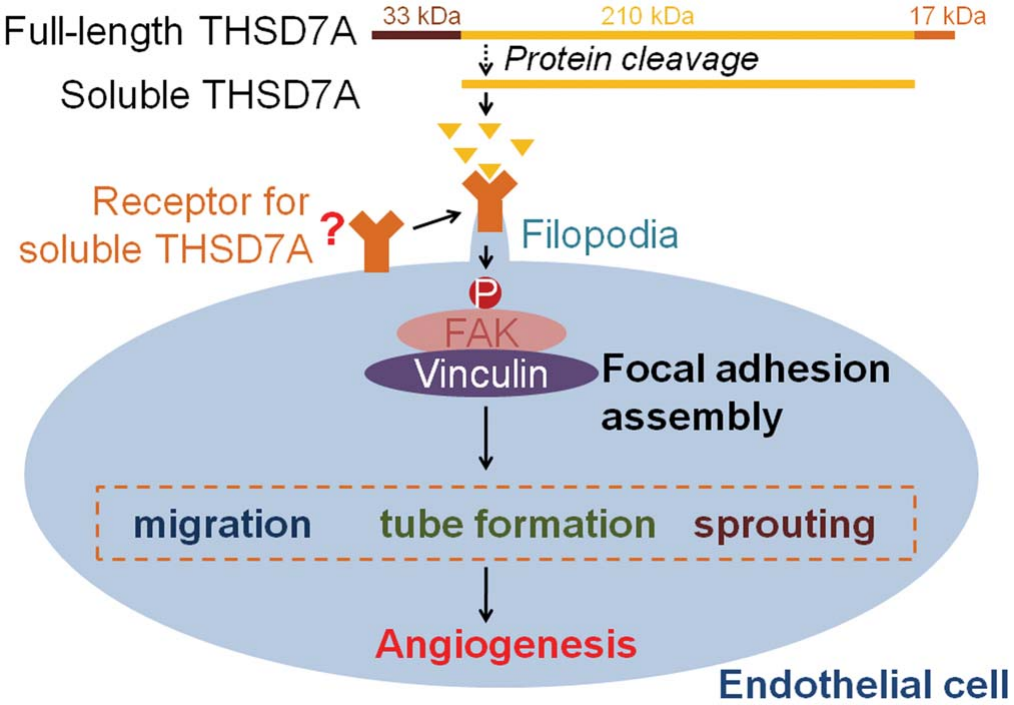
Hypothesized mechanism for the role of soluble THSD7A in angiogenesis. Soluble THSD7A is released from full-length THSD7A. In our hypothesis, soluble THSD7A binds receptor and triggers filopodia formation and focal adhesion assembly to promote cell migration, tube formation and sprout formation via a FAK-involved manner.

In this study, we identified a soluble form of THSD7A that is produced by cells of both endothelial and neuronal origins. This soluble THSD7A is a N-glycoprotein that can promote endothelial cell migration, tube formation and sprouting. Soluble THSD7A also increases SIV branching and alters vascular patterning in the zebrafish embryo. Furthermore, we determined that these effects may be induced via regulation of filopodia formation and focal adhesion assembly in endothelial cells. Finally, FAK-dependent signaling pathways were shown to be activated by soluble THSD7A, which suggests that soluble THSD7A may be involved in the regulation of focal adhesions during angiogenic cell migration. Addition of anti-sTHSD7A antibody blocked the angiogenic effects of soluble THSD7A and demonstrated its specificity. Based on these data, we have developed a model for the potential role of soluble THSD7A in angiogenesis (Figure 8).

Based on bioinformatic prediction, THSD7A contains several domains and motifs that might regulate angiogenesis. For example, THSD7A contains at least ten thrombospondin type 1 repeats (TSRs). TSRs are found in thrombospondin-1, thrombospondin-2, semaphorins, UNC-5 and F-spondin, most of which are expressed in the developing nervous system and have functional roles in neural cell guidance and growth cone migration [25]. In addition, THSD7A contains six extracellular WSXW motifs, which are located in the TSRs. The WSXW motif in thrombospondin-1 has been shown to bind and activate latent TGF-β [26]–[27]. It can also interact with fibronectin and regulate fibronectin-mediated adhesion [28] and promote cell adhesion by binding to heparin and heparin sulfate [29]. The WSXW motif is also critical in promoting cell spreading and FAK phosphorylation [30]. Therefore, these functional domains/motifs in THSD7A might enable it to regulate endothelial cell migration.

THSD7A was initially predicted to be a type I membrane protein. Our data support this prediction and further show that THSD7A releases part of its amino-terminal region into the extracellular environment (Figure 2,3). The level of soluble THSD7A in the cultured medium is reduced after blocking N-glycosylation, suggesting that N-glycosylation is important for THSD7A trafficking. In addition to the full-length form (260 kDa), THSD7A appears to have three other isoforms: the functional soluble form (210 kDa; Figure 3C, arrow), the amino-terminal form (33 kDa; Figure 3C, arrowhead) and the membrane-anchored form (17 kDa; Figure 2, arrow). Based on our data, we suggest that full-length THSD7A is modified first by N-glycosylation, which leads to anchoring in the plasma membrane. A yet-to-be-identified process may cause the amino-terminal region of THSD7A to be released into the extracellular environment. Second, the extracellular region of THSD7A is likely cleaved at two sites: in the region between the IDS9 and the CTE epitopes and within the sTHSD7A epitope site, thus generating the functional soluble form (Figure 8). In addition, the 33 kDa amino-terminal form of THSD7A includes a region that contains the predicted signal peptide (the first 47 amino acids from amino terminus, ∼13 kDa).

The nature of the processing scheme that releases soluble THSD7A is an interesting question. Many proteins are modified by proteolysis to regulate their function and activity. For example, mature VEGF-C is cleaved and released from a VEGF-C precursor to induce endothelial cell migration [31]. Similarly, TGF-b must be activated by cleavage of the latent TGF-b before it can perform its diverse biological functions, including immunoregulation and control of proliferation [32]. The release of soluble THSD7A is protease-dependent (Figure S1), but the specific protease that cleaves THSD7A is still unknown.

Endothelial cell migration is a complex process containing several steps: sensing the motile stimuli by filopodia, extending protrusions at the leading edge, assembly of focal adhesions to attach the protrusions to ECM, formation of stress fibers, and recycling of the adhesive and signaling components [1], [5], [10], [22]. During this process, endothelial tip cells are key players in sensing angiogenic motility stimuli [5]. Previous studies have indicated that angiogenic sprouts are enriched for migratory filopodia [5], [33]. In this study, our data demonstrate that soluble THSD7A can promote endothelial cell migration, tube formation, sprouting (Figure 4) and filopodia formation (Figure 6B). These results are further supported by the observation that soluble THSD7A affects angiogenic branching in the developing zebrafish (Figure 5). These data all indicate that soluble THSD7A is a pro-angiogenic protein that is important for filopodia formation during endothelial cell migration.

The effect of soluble THSD7A appears to occur in the initial steps of migration. Soluble THSD7A promotes filopodia formation, which is consistent with the roles of many angiogenic proteins, such as VEGF-A and VEGF-C [33]–[34]. Besides filopodia formation, we also revealed that soluble THSD7A can promote FAK/vinculin assembly (Figure 6C–D). Assembly of the focal adhesion complex (including FAK, vinculin, paxillin and others) is an important step in stabilizing the adherence of filopodial protrusions to the ECM. Focal adhesions also link the cytoskeleton to integrins and support actin-based motility [22]. As shown in our data, soluble THSD7A significantly increases the phosphorylation level of FAK (Figure 6E). This is an important step that precedes downstream signal transduction [24]. After FAK is activated by phosphorylation, vinculin is recruited to the FAK carboxyl-terminal focal adhesion targeting site (FAT) and modulating cell migration, actin polymerization and mitogen-activated protein kinase (MAPK, including Erk1/2 and P38) signaling [12]. As shown in Figure 6E, we found the phosphorylation levels of Erk1/2 and P38 were slightly increased with soluble THSD7A treatment. MAPKs have been shown to be stimulated by growth factors such as VEGF, fibroblast growth factor, epidermal growth factor and platelet-derived growth factor and are known to play an essential role in the signaling pathways linking diverse extracellular signals to vital cellular processes [35]–[37]. These growth factors activate Erk through the Ras-Raf-MEK1/2-Erk signaling module [38], which then phosphorylates myosin light chain kinase (MLCK) and FAK to regulate membrane protrusion and focal adhesion dynamics [35], [39]. P38, another member of the MAPK family, is well known for its involvement in inflammation, apoptosis and cell differentiation [40]. P38 has been shown to be involved in cell migration through regulation of the phosphorylation of heat shock protein 27 (HSP27) [41]–[42] and activation of MAPK-activated protein kinase 2/3 [43]. Together with FAK phosphorylation, our study indicates that Erk1/2 and P38 might also partially contribute to the downstream cell migration signaling pathways induced by soluble THSD7A.

It is also unclear what the potential receptor for soluble THSD7A might be. As previous studies have indicated, integrins are a diverse glycoprotein family that can recruit focal adhesion components and then link to the cytoskeleton to regulate cell migration [44]–[45]. Collagen, fibronectin, vitronectin, thrombospondin-1, thrombin and angiostatin are reported to interact with integrins to regulate endothelial cell migration and angiogenesis [45]–[46]. There are similarities between the effects of integrins and the effects of soluble THSD7A on endothelial cells. Thus, integrins are possible receptor candidates for soluble THSD7A. Integrins recognize an Arg-Gly-Asp (RGD) motif on many adhesion proteins, including fibronectin, vitronectin, thrombospondin, laminin and fibrinogen [47]. Interestingly, there is one RGD motif located in the extracellular region of THSD7A (Figure 1A), suggesting that integrins (for example, the endothelial-specific αvβ3 integrin) might interact with this RGD motif on THSD7A. Thus, it would be worthwhile to investigate the interaction between soluble THSD7A and integrins in future studies. On the other hand, the well-studied protein VEGFR2 and its downstream signaling pathways are also critical in regulating angiogenesis, so we cannot not rule out the possibility that soluble THSD7A may interact with VEGFR2 or its co-receptors [48]–[49].

Data from our previous studies have illustrated that THSD7A is a neural protein in zebrafish and is expressed in the placenta vasculature in human. This suggests the THSD7A expression pattern from zebrafish to human may be different, and it may play different roles in various organs and developmental stages. Nevertheless, the link and interplay between the membrane-associated and soluble forms of THSD7A and their effects on endothelial cell migration remain unclear [15]–[16]. In the present study, our results support the hypothesis that THSD7A may have a neuronal origin and that soluble THSD7A is the functional form that promotes endothelial cell migration *in vitro* and *in vivo*. Several neuronal-secreted axon guidance molecules, including the netrins, slits, semaphorins and ephrins, have been implicated in endothelial cell migration and tube formation and are now called neuroangiogenic factors [50]. It has been shown that netrin-1 is secreted from the ventral midline of the central nervous system, where it regulates axon guidance [51]. However, netrin-1 treatment causes retraction of the filopodia of endothelial tip cells and is thought to be a repulsive vessel guidance cue [52]. Slit-2, another neural protein, is expressed in the nervous system midline and serves as a repulsive axon guidance cue [51]. Conversely, Slit-2 treatment stimulates endothelial cell migration *in vitro* and tumor angiogenesis *in vivo* [53]. In conclusion, in light of our current findings and previous results, we propose that THSD7A is a novel neuroangiogenic factor.

## Supporting Information

Figure S1 **The release of soluble THSD7A is protease-dependent.** **A.** *THSD7A*-transfected HEK293T cells were treated with complete protease inhibitor (C), Benzamidine (B), Leupeptin (L) and Pepstatin A (P). Untreated cells (No) served as a control. Cell lysates and cultured medium were subjected to Western blot with anti-sTHSD7A antibody. GAPDH served as a loading control. **B.** Two proteases, thrombin and caspase 1, are predicted to cleave the full-length THSD7A. **C.** *THSD7A*-transfected HEK293T cells were treated with complete protease inhibitor (C), hirudin (H), caspase 1 inhibitor I (I) or no treatment (No). Cell lysates and cultured medium were subjected to Western blot with anti-sTHSD7A antibody. GAPDH served as a loading control.
(TIF)

Data S1 **Materials and Methods.**
(DOC)

## References

[1] Munoz-Chapuli R, Quesada AR, Angel Medina M (2004). Angiogenesis and signal transduction in endothelial cells.. Cell Mol Life Sci.

[2] Hoeben A, Landuyt B, Highley MS, Wildiers H, Van Oosterom AT (2004). Vascular endothelial growth factor and angiogenesis.. Pharmacol Rev.

[3] Adams RH, Alitalo K (2007). Molecular regulation of angiogenesis and lymphangiogenesis.. Nat Rev Mol Cell Biol.

[4] Horowitz A, Simons M (2008). Branching morphogenesis.. Circ Res.

[5] De Smet F, Segura I, De Bock K, Hohensinner PJ, Carmeliet P (2009). Mechanisms of vessel branching: filopodia on endothelial tip cells lead the way.. Arterioscler Thromb Vasc Biol.

[6] Jakobsson L, Franco CA, Bentley K, Collins RT, Ponsioen B (2010). Endothelial cells dynamically compete for the tip cell position during angiogenic sprouting.. Nat Cell Biol.

[7] Hellstrom M, Phng LK, Hofmann JJ, Wallgard E, Coultas L (2007). Dll4 signalling through Notch1 regulates formation of tip cells during angiogenesis.. Nature.

[8] Suchting S, Freitas C, le Noble F, Benedito R, Breant C (2007). The Notch ligand Delta-like 4 negatively regulates endothelial tip cell formation and vessel branching.. Proc Natl Acad Sci U S A.

[9] Galbraith CG, Yamada KM, Galbraith JA (2007). Polymerizing actin fibers position integrins primed to probe for adhesion sites.. Science.

[10] Mattila PK, Lappalainen P (2008). Filopodia: molecular architecture and cellular functions.. Nat Rev Mol Cell Biol.

[11] Braren R, Hu H, Kim YH, Beggs HE, Reichardt LF (2006). Endothelial FAK is essential for vascular network stability, cell survival, and lamellipodial formation.. J Cell Biol.

[12] Parsons JT (2003). Focal adhesion kinase: the first ten years.. J Cell Sci.

[13] Miranti CK, Brugge JS (2002). Sensing the environment: a historical perspective on integrin signal transduction.. Nat Cell Biol.

[14] Parsons JT, Martin KH, Slack JK, Taylor JM, Weed SA (2000). Focal adhesion kinase: a regulator of focal adhesion dynamics and cell movement.. Oncogene.

[15] Wang CH, Su PT, Du XY, Kuo MW, Lin CY (2010). Thrombospondin type I domain containing 7A (THSD7A) mediates endothelial cell migration and tube formation.. J Cell Physiol.

[16] Wang CH, Chen IH, Kuo MW, Su PT, Lai ZY (2011). Zebrafish Thsd7a is a neural protein required for angiogenic patterning during development.. Dev Dyn.

[17] Davis GE, Saunders WB (2006). Molecular balance of capillary tube formation versus regression in wound repair: role of matrix metalloproteinases and their inhibitors.. J Investig Dermatol Symp Proc.

[18] Stratman AN, Davis MJ, Davis GE (2011). VEGF and FGF prime vascular tube morphogenesis and sprouting directed by hematopoietic stem cell cytokines.. Blood.

[19] Nicoli S, De Sena G, Presta M (2009). Fibroblast growth factor 2-induced angiogenesis in zebrafish: the zebrafish yolk membrane (ZFYM) angiogenesis assay.. J Cell Mol Med.

[20] Sinha Roy R, Soni S, Harfouche R, Vasudevan PR, Holmes O (2010). Coupling growth-factor engineering with nanotechnology for therapeutic angiogenesis.. Proc Natl Acad Sci U S A.

[21] Serbedzija GN, Flynn E, Willett CE (1999). Zebrafish angiogenesis: a new model for drug screening.. Angiogenesis.

[22] Lamalice L, Le Boeuf F, Huot J (2007). Endothelial cell migration during angiogenesis.. Circ Res.

[23] Vainionpaa N, Kikkawa Y, Lounatmaa K, Miner JH, Rousselle P (2006). Laminin-10 and Lutheran blood group glycoproteins in adhesion of human endothelial cells.. Am J Physiol Cell Physiol.

[24] Sieg DJ, Hauck CR, Ilic D, Klingbeil CK, Schaefer E (2000). FAK integrates growth-factor and integrin signals to promote cell migration.. Nat Cell Biol.

[25] Adams JC, Tucker RP (2000). The thrombospondin type 1 repeat (TSR) superfamily: diverse proteins with related roles in neuronal development.. Dev Dyn.

[26] Murphy-Ullrich JE, Poczatek M (2000). Activation of latent TGF-beta by thrombospondin-1: mechanisms and physiology.. Cytokine Growth Factor Rev.

[27] Young GD, Murphy-Ullrich JE (2004). The tryptophan-rich motifs of the thrombospondin type 1 repeats bind VLAL motifs in the latent transforming growth factor-beta complex.. J Biol Chem.

[28] Sipes JM, Guo N, Negre E, Vogel T, Krutzsch HC (1993). Inhibition of fibronectin binding and fibronectin-mediated cell adhesion to collagen by a peptide from the second type I repeat of thrombospondin.. J Cell Biol.

[29] Guo NH, Krutzsch HC, Negre E, Vogel T, Blake DA (1992). Heparin- and sulfatide-binding peptides from the type I repeats of human thrombospondin promote melanoma cell adhesion.. Proc Natl Acad Sci U S A.

[30] Sipes JM, Krutzsch HC, Lawler J, Roberts DD (1999). Cooperation between thrombospondin-1 type 1 repeat peptides and alpha(v)beta(3) integrin ligands to promote melanoma cell spreading and focal adhesion kinase phosphorylation.. J Biol Chem.

[31] Joukov V, Sorsa T, Kumar V, Jeltsch M, Claesson-Welsh L (1997). Proteolytic processing regulates receptor specificity and activity of VEGF-C.. EMBO J.

[32] Lawrence DA (2001). Latent-TGF-beta: an overview.. Mol Cell Biochem.

[33] Gerhardt H, Golding M, Fruttiger M, Ruhrberg C, Lundkvist A (2003). VEGF guides angiogenic sprouting utilizing endothelial tip cell filopodia.. J Cell Biol.

[34] Benest AV, Harper SJ, Herttuala SY, Alitalo K, Bates DO (2008). VEGF-C induced angiogenesis preferentially occurs at a distance from lymphangiogenesis.. Cardiovasc Res.

[35] Huang C, Jacobson K, Schaller MD (2004). MAP kinases and cell migration.. J Cell Sci.

[36] Tangkijvanich P, Santiskulvong C, Melton AC, Rozengurt E, Yee HF (2002). p38 MAP kinase mediates platelet-derived growth factor-stimulated migration of hepatic myofibroblasts.. J Cell Physiol.

[37] Robinson MJ, Cobb MH (1997). Mitogen-activated protein kinase pathways.. Curr Opin Cell Biol.

[38] Seger R, Krebs EG (1995). The MAPK signaling cascade.. FASEB J.

[39] Klemke RL, Cai S, Giannini AL, Gallagher PJ, de Lanerolle P (1997). Regulation of cell motility by mitogen-activated protein kinase.. J Cell Biol.

[40] Ono K, Han J (2000). The p38 signal transduction pathway: activation and function.. Cell Signal.

[41] Hedges JC, Dechert MA, Yamboliev IA, Martin JL, Hickey E (1999). A role for p38(MAPK)/HSP27 pathway in smooth muscle cell migration.. J Biol Chem.

[42] Rousseau S, Houle F, Landry J, Huot J (1997). p38 MAP kinase activation by vascular endothelial growth factor mediates actin reorganization and cell migration in human endothelial cells.. Oncogene.

[43] McLaughlin MM, Kumar S, McDonnell PC, Van Horn S, Lee JC (1996). Identification of mitogen-activated protein (MAP) kinase-activated protein kinase-3, a novel substrate of CSBP p38 MAP kinase.. J Biol Chem.

[44] Hood JD, Cheresh DA (2002). Role of integrins in cell invasion and migration.. Nat Rev Cancer.

[45] Silva R, D'Amico G, Hodivala-Dilke KM, Reynolds LE (2008). Integrins: the keys to unlocking angiogenesis.. Arterioscler Thromb Vasc Biol.

[46] Hodivala-Dilke KM, Reynolds AR, Reynolds LE (2003). Integrins in angiogenesis: multitalented molecules in a balancing act.. Cell Tissue Res.

[47] Ruoslahti E (1996). RGD and other recognition sequences for integrins.. Annu Rev Cell Dev Biol.

[48] Somanath PR, Malinin NL, Byzova TV (2009). Cooperation between integrin alphavbeta3 and VEGFR2 in angiogenesis.. Angiogenesis.

[49] Masson-Gadais B, Houle F, Laferriere J, Huot J (2003). Integrin alphavbeta3, requirement for VEGFR2-mediated activation of SAPK2/p38 and for Hsp90-dependent phosphorylation of focal adhesion kinase in endothelial cells activated by VEGF.. Cell Stress Chaperones.

[50] Park JA, Choi KS, Kim SY, Kim KW (2003). Coordinated interaction of the vascular and nervous systems: from molecule- to cell-based approaches.. Biochem Biophys Res Commun.

[51] Eichmann A, Makinen T, Alitalo K (2005). Neural guidance molecules regulate vascular remodeling and vessel navigation.. Genes Dev.

[52] Lu X, Le Noble F, Yuan L, Jiang Q, De Lafarge B (2004). The netrin receptor UNC5B mediates guidance events controlling morphogenesis of the vascular system.. Nature.

[53] Wang B, Xiao Y, Ding BB, Zhang N, Yuan X (2003). Induction of tumor angiogenesis by Slit-Robo signaling and inhibition of cancer growth by blocking Robo activity.. Cancer Cell.

