# Supplementary figures and images for: Soluble THSD7A Is an N-Glycoprotein That Promotes Endothelial Cell Migration and Tube Formation in Angiogenesis
Source: PLoS One. 2011 Dec 14;6(12):e29000. doi: 10.1371/journal.pone.0029000 (PMC3237571; doi:10.1371/journal.pone.0029000)

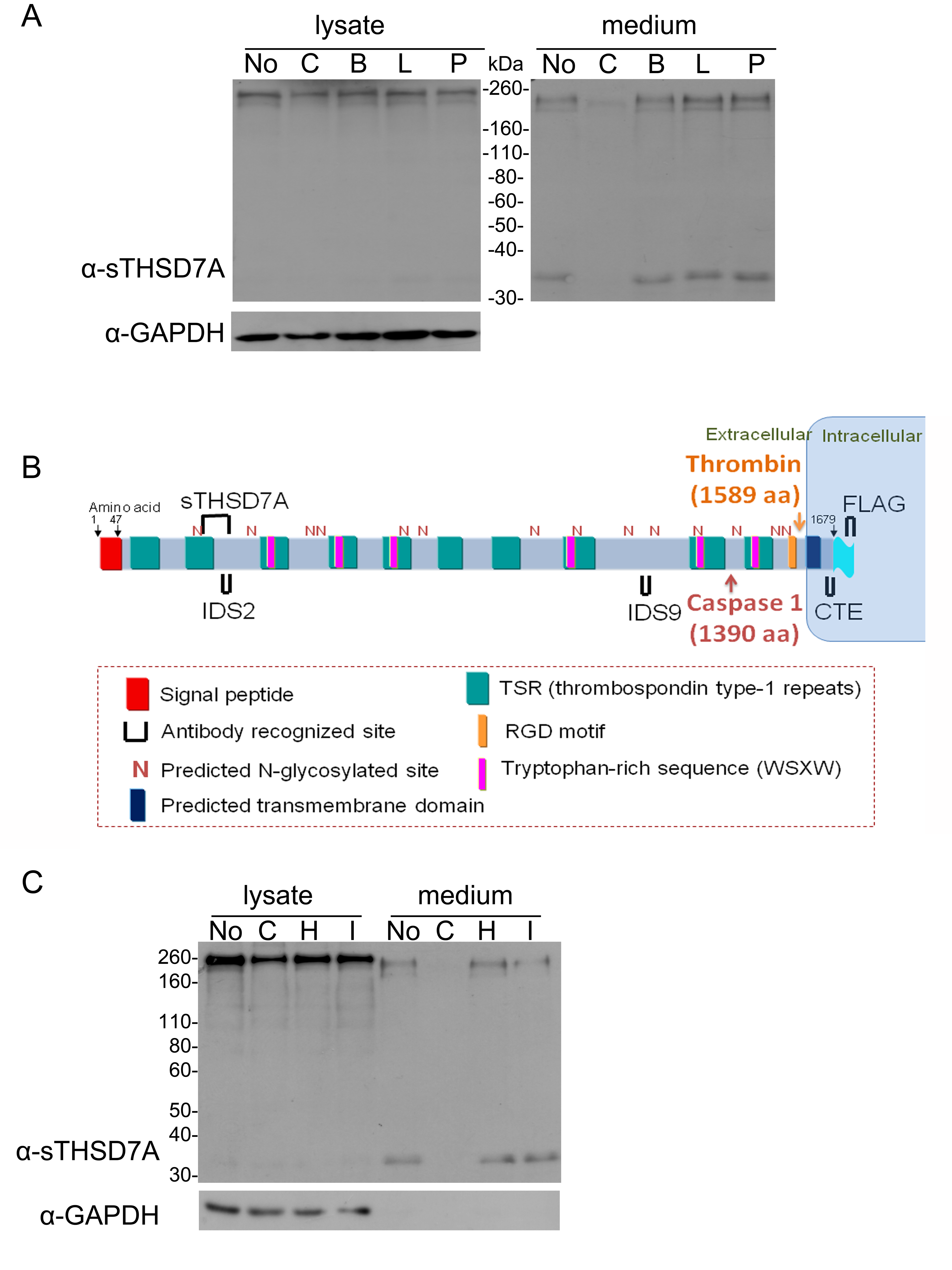

Supplement: Figure S1 — The release of soluble THSD7A is protease-dependent. A. THSD7A-transfected HEK293T cells were treated with complete protease inhibitor (C), Benzamidine (B), Leupeptin (L) and Pepstatin A (P). Untreated cells (No) served as a control. Cell lysates and cultured medium were subjected to Western blot with anti-sTHSD7A antibody. GAPDH served as a loading control. B. Two proteases, thrombin and caspase 1, are predicted to cleave the full-length THSD7A. C. THSD7A-transfected HEK293T cells were treated with complete protease inhibitor (C), hirudin (H), caspase 1 inhibitor I (I) or no treatment (No). Cell lysates and cultured medium were subjected to Western blot with anti-sTHSD7A antibody. GAPDH served as a loading control. (TIF) [file pone.0029000.s001.tif]
