# Supplementary material for: Soluble THSD7A Is an N-Glycoprotein That Promotes Endothelial Cell Migration and Tube Formation in Angiogenesis
Source: PLoS One. 2011 Dec 14;6(12):e29000. doi: 10.1371/journal.pone.0029000 (PMC3237571; doi:10.1371/journal.pone.0029000)
Supplement: Data S1 — Materials and Methods. (DOC) [file pone.0029000.s002.doc]

**Supplementary data**

**Materials and Methods**

**Ethics Statement**

All of the zebrafish-use protocols in this research were reviewed and approved by the Institutional Animal Care and Use Committee of National Tsing Hua University (IRB Approval NO. 09507).

**Zebrafish stocks**

The Tg(kdr:EGFP)^y1^ transgenic strain was used in the study [1]. Embryos were raised at 28.5°C and staged as described [2].

**Zebrafish angiogenesis assay**

The zebrafish angiogenesis assay was performed as described previously [3-4]. Briefly, zebrafish (50 hours post fertilization, hpf) were anesthetized in 65 mM tricaine and 65 mM isoflurane.[5] We then injected 9.2 nl of soluble THSD7A (~ 0.69 ng) or control medium into the yolk sac. Zebrafish without any injection comprised the normal group. After incubating at 28.5°C for 24 hours after the injection, both sides of the subintestinal vessel (SIV) in the yolk sac were monitored by inverted fluorescence microscopy and the branch point number was calculated.

**Human umbilical vein endothelial cell (HUVEC) isolation**

The primary HUVEC was isolated from termed placenta with signed consent from patients under protocols approved by the Institutional Review Board of Human Subjects Research Ethics Committee of Mackay Memorial Hospital (10MMHIS135 and MMH-I-S-137), Taipei City, Taiwan. HUVECs were isolated from fresh umbilical cord that displayed no clamp marks or needle holes as previously described [6]. The umbilical vein was slowly washed twice with 10 ml PBS to remove residual blood and clots, then filled with collagenase and sealed using hemostats. The vein was then incubated for 7 minutes in 37°C PBS, after which the vein endothelial cell sheets released from the inner lining of the blood vessel. The eluate was collected into a sterile 50 ml tube after the hemostats were removed, and the collagenase activity was terminated by Dulbecco's Modified Eagle's Medium (DMEM) supplemented with 10*%* Fetal Bovine Serum (FBS). The eluate was then centrifuged at 200 ×g for 5 minutes. The cell pellet was then suspended in M200 (Invitrogen, Carlsbad, CA) supplemented with Low Serum Growth Supplement (LSGS) (Invitrogen, containing 2% FBS, 3 ng/ml basic fibroblast growth factor, 10 μg/ml heparin, 1 μg/ml hydrocortisone, and 10 ng/ml epidermal growth factor) and seeded in a 0.2% gelatin-coated flask.

**Cell culture**

The human embryonic kidney cell line 293T (HEK293T, CRL-11268) was purchased from GenDiscovery Biotechnology Inc. The SH-SY5Y human neuroblastoma cell line (CRL-2266, originally purchased from the American Type Culture Collection, Manassas, VA, USA) was a generous gift from Dr. HH Shen of  Industrial Technology Research Institute, Hsinchu, Taiwan. HEK293T cells and SH-SY5Y cells were maintained in DMEM supplemented with 10% FBS, 100 μg/ml penicillin, 100 μg/ml streptomycin and 2.5 μg/ml amphotericin B. After *THSD7A* transfection, HEK293T cells were maintained in serum-free DMEM.

**Construction of human full-length *THSD7A* plasmid**

The full-length *THSD7A* coding sequence was isolated from human placental cDNA. *THSD7A* was amplified using polymerase chain reaction (PCR) and primers containing SacII and XhoI sites. The sequences of the cloning primers are 5’-AAA CCG CGG ATG GGG CTG CAA GCC AGG-3’ and 5’- AAA CTC GAG TTT GTC GGC ATC TCC ATC ATA G-3’. The PCR product was subsequently cloned into the pCMVTag4 expression vector (Stratagene, La Jolla, CA), generating a recombinant protein with a FLAG-tag fused to the C-terminus of full-length THSD7A (Figure 1A). All sequence and molecular weight prediction and was analyzed using the ExPASy proteomic server.

**Preparation of THSD7A-specific antibodies**

We purified three THSD7A-specific custom antibodies (anti-IDS2, anti-IDS9 and anti-CTE) in our laboratory (Figure 1A). The IDS2, IDS9 and CTE peptides and the rabbit antisera against these peptides were synthesized and produced, respectively, by LTK BioLaboratory. Peptides were conjugated to cyanogen bromide-activated sepharose (Sigma) in coupling buffer (0.2 M sodium carbonate, 0.5 M sodium chloride, pH 8.3) at 4°C overnight. Rabbit antisera were applied to the sepharose-bound peptides and incubated at 4°C overnight. After incubation with the rabbit antisera, the sepharose were washed with 6 volumes of coupling buffer and bound antibodies were eluted with elution buffer (0.1 M sodium acetate, 0.5 M sodium chloride, pH 4) at 4°C. The eluted antibodies were neutralized immediately with 1 M Tris-HCl (pH 7.5) after elution. The specificity of purified antibodies was checked by enzyme-linked immunosorbent assay (ELISA) and Western blot. For clarity, the commercial anti-THSD7A antibody (HPA000923, Sigma) is referred to as anti-sTHSD7A in this article. Anti-sTHSD7A recognizes 116 amino acids of THSD7A, including the IDS2 region (Figure 1A).

**Cell transfection and soluble THSD7A preparation**

HEK293T cells were transiently transfected with the *THSD7A* expression vector or empty pCMVTag4 using jetPEI reagent (Polyplus) according to the manufacturer's protocol. Briefly, HEK293T cells were seeded at 2x10^6^ cells per 10 cm culture dish and incubated overnight at 37°C. Then, the cells were incubated with the jetPEI-DNA complex in serum-free DMEM, which was exchanged for fresh serum-free DMEM after 8 hours. Cells and cultured medium were harvested 48 hours after transfection for further analysis. To collect soluble TDSD7A, the cultured medium of *THSD7A*-transfected HEK293T cells was collected and centrifuged to pellet down any suspended cells. The supernatants were centrifuged through an Amicon membrane filter with a 100-kDa cutoff (Millipore) to yield a ~100x concentrated cultured medium that was enriched for proteins >100 kDa. The cultured medium of empty vector-transfected HEK293T cells was subjected to the same process and served as a negative control. The final concentration of concentrated soluble THSD7A was ~75.5 ng/ml which was detected by ELISA.

**Subcellular fractionation**

Cytosolic (C), nuclear (N) and membrane (M) fractions of *THSD7A*-transfected HEK293T cells were prepared using a CNM compartment protein extraction kit (BioChain Institute Inc., Hayward, CA) according to the manufacturer’s instructions. Briefly, *THSD7A*-transfected HEK293T cells (1x10^7^) were harvested and washed with PBS. The cell pellet was homogenized in 1 ml buffer C plus a mixture of protease inhibitors, passed through a 27.5 gauge needle 50 times, rotated for 20 minutes at 4°C, then centrifuged at 16000 ×g for 20 minutes at 4°C. The resulting supernatant comprised the cytosolic (C) fraction. The insoluble pellet was washed with 2 ml of buffer W and resuspended in 500 μl of buffer N, then incubated at 4°C for 20 minutes. Subsequent centrifugation at 16000 ×g for 20 minutes at 4°C resulted in a supernatant that contained the nuclear (N) fraction. Then, 500 μl of buffer M was used to resuspend the pellet, and the sample was rotated at 4°C for 20 minutes and centrifuged at 16000 ×g for 20 minutes 4°C. The resulting supernatant contained the membrane proteins (M) and was extracted. As controls, calnexin served as plasma membrane marker and GAPDH served as cytoplasm marker. Annexin V served as cytoplasm and plasma membrane marker.

**Tunicamycin treatment**

Transfected HEK293T cells were treated with or without 5 μg/ml tunicamycin (Sigma) in supplemented DMEM at 37°C. After 48 hours incubation, cell lysates and cultured media were harvested and subjected to Western blot analysis.

**Soluble THSD7A treatment**

HUVECs were trypsinized and seeded to 80-90% confluence in a type I collagen-coated flask. Three hours after seeding, HUVEC cultured media were changed to M200 supplemented with 0.1% BSA for overnight starvation. The next day, the medium was replaced with 20% soluble THSD7A or control medium in fresh M200 supplemented with 0.1% BSA. After 15 minutes of incubation, HUVECs were lysed with RIPA buffer (Pierce) containing a Protease Inhibitor Cocktail (Roche), Sodium Fluoride and Sodium Orthovanadate, and subjected to Western blot analysis.

**SDS-PAGE and Western blot**

HEK293T cell lysate and cultured media proteins were separated on 4-12% Bis-Tris gradient gels using the NuPAGE electrophoresis system (Invitrogen) and MES running buffer. Soluble THSD7A- and vector-treated HUVEC lysates were separated using 8% sodium dodecyl sulfate polyacrylamide gels (SDS-PAGE). Proteins separated by NuPAGE or SDS-PAGE were transferred to PVDF membranes. Non-specific antibody binding was blocked with 3% BSA in TBS buffer containing 0.5% Tween 20; the membranes were then incubated with primary antibody overnight at 4°C. Species-specific Horseradish Peroxidase (HRP)-conjugated secondary antibodies were used for detection of primary antibodies, and visualization was achieved with a chemiluminescent substrate. The anti-FAK pY397 antibody was from Invitrogen. The anti-Akt pS473, P38 pT180/R182 and Erk1/2 pT202/Y204 antibodies were from Cell Signaling. The anti-glyceraldehyde 3-phosphate dehydrogenase (GAPDH) antibody was from Santa Cruz. The anti-FLAG-tag M2 antibody was from Sigma.

**HUVEC adhesion, filopodia formation, and vinculin and FAK pY397 distribution assays**

Glass coverslips were coated with type I collagen (10 μg/ml) and incubated 1 hour at 37°C. Poly-L-lysine (PLL, 30 μg/ml) was coated on glass coverslips overnight at room temperature and served as non-extracellular matrix control. HUVECs were trypsinized, and 3000 cells were seeded per coverslip in M200 containing 20% soluble THSD7A or control culture medium. After 50 minutes incubation at 37°C, the adherent HUVECs were fixed with 4% paraformaldehyde. Subsequently, we performed immunocytochemistry on the adherent HUVECs using anti-vinculin (Sigma) or anti-FAK pY397 antibodies (Invitrogen), phalloidin and Hoechst. The number of HUVECs in at least three independent fields was counted at 100x magnification. Vinculin distribution, FAK pY397 distribution and filopodia formation were analyzed in at least 30 HUVECs at 630x magnification using a confocal microscope (LSM510 Meta, Zeiss). Each condition was repeated independently at least three times.

**Migration Assay**

HUVEC motility was assayed using Transwell (BD) polycarbonate filter inserts (8 μm pore size). The filter surface was coated with type I collagen (BD). Soluble THSD7A or control medium was added to a final concentration of 3% or 15% in M200 with 0.1% BSA in the lower well, and 5x10^4^ HUVECs were loaded in M200 with 0.1% BSA into the upper well. After 4 hour incubation at 37^o^C, cells were fixed with methanol and stained with Hoechst. The number of cells that migrated to the lower side of filter was quantified using images captured at 100x magnification by inverted fluorescence microscopy (Nikon TE2000E) with a cooled CCD (Evolution VF, MediaCybernetics, Bethesda, MD) and processed using the program Extended Depth of Field in the Image-pro plus AMS software. Each sample was analyzed in three to five random fields and independently repeated at least three times.

**Two-dimensional tube formation assay**

Basement membrane matrix (Matrigel, BD Biosciences) was thawed at 4^o^C, and 200 μl was used to coat each well of a 48-well plate. The plate was incubated 30 minutes at 37^o^C to polymerize the Matrigel. HUVECs (5.5x10^4^ cells/100 μl) were suspended in supplemented M200 containing 20% soluble THSD7A or control medium, plated on a Matrigel-coated well and allowed to adhere and migrate for 3 hours at 37^o^C. Tube length was quantified at using 40x magnification pictures that were captured and processed as described above. Each sample was assayed in two to three random fields and independently repeated three times.

**Three-dimensional tube formation assay**

Feeding media and 2.5 mg/ml type I collagen matrices were prepared as described [7]. HUVECs were trypsinized and suspended in feeding media. Then, 1x10^5^ cells were seeded into 96 well plates and incubated for 3 hours at 37^o^C. After cells adhered to the bottom of the dish, the feeding medium was removed, 58 μl of 2.5 mg/ml collagen type 1 solution was immediately added to each well, and the plate was incubated for 30 minutes at 37^o^C to polymerize the collagen matrixice. Then 200 μl feeding medium mixed with 25% soluble THSD7A or control medium was then added to each well, and the plates were incubated at 37^o^C. After 24 hours, HUVECs were fixed with 4% paraformaldehyde for at least 4 hours and stained with 1% toluidine blue. After destaining with deionized distilled water, five random views were captured at 100x magnification by inverted fluorescence microscopy (Nikon TE2000E), and the number of tubes was calculated.

**Antibody neutralization assay**

Soluble THSD7A preparation, migration assay, filopodia formation and vinculin distribution assay were described above. The concentrated soluble THSD7A was incubated with 80 ng/ml anti-sTHSD7A antibody for 1 hour at 37^o^C and then performed the functional assays. The concentrated soluble THSD7A without antibody addition and the control medium were also incubated 1 hour at 37^o^C.

**Protease inhibitor treatment**

*THSD7A*-transfected HEK293T cells were incubated with protease inhibitors including 1x complete protease inhibitor (EDTA-containing, inhibitor of serine, cysteine and metallo-protease, purchased from Roche), 1 μg/ml leupeptin (inhibitor of serine and cysteine proteases), 1 μg/ml pepstatin A (inhibitor of aspartic proteases), 1 mM benzamidine (inhibitor of trypsin, trypsin-like enzymes and serine proteases), 20 μM hirudin (inhibitor of thrombin) or 20 μM caspase 1 inhibitor I for total 24 hours at 37^o^C and added these protease inhibitors every 12 hours. The supernatants were centrifuged through an Amicon membrane filter with a 3 kDa cutoff (Millipore) to yield a ~100x concentrated cultured medium and subjected to Western blot with cell lysates. The protease inhibitor prediction is analyzed by ExPASy “PeptideCutter”.

**Statistical analysis**

Student’s t-test was used to determine statistical significance. All *P* values were derived from at least three independent experiments. A value of *P*<0.05 was accepted as statistically significant. Data are presented as the mean ± SD. **P*<0.05, ***P*<0.01 were used to indicate statistical significance.

**Literature cited**

1. Lawson ND, Weinstein BM (2002) Arteries and veins: making a difference with zebrafish. Nat Rev Genet 3: 674-682.

2. Kimmel CB, Ballard WW, Kimmel SR, Ullmann B, Schilling TF (1995) Stages of embryonic development of the zebrafish. Dev Dyn 203: 253-310.

3. Sinha Roy R, Soni S, Harfouche R, Vasudevan PR, Holmes O, et al. (2010) Coupling growth-factor engineering with nanotechnology for therapeutic angiogenesis. Proc Natl Acad Sci U S A 107: 13608-13613.

4. Nicoli S, De Sena G, Presta M (2009) Fibroblast growth factor 2-induced angiogenesis in zebrafish: the zebrafish yolk membrane (ZFYM) angiogenesis assay. J Cell Mol Med 13: 2061-2068.

5. Huang WC, Hsieh YS, Chen IH, Wang CH, Chang HW, et al. (2010) Combined use of MS-222 (tricaine) and isoflurane extends anesthesia time and minimizes cardiac rhythm side effects in adult zebrafish. Zebrafish 7: 297-304.

6. Wang CH, Su PT, Du XY, Kuo MW, Lin CY, et al. (2010) Thrombospondin type I domain containing 7A (THSD7A) mediates endothelial cell migration and tube formation. J Cell Physiol 222: 685-694.

7. Koh W, Stratman AN, Sacharidou A, Davis GE (2008) In vitro three dimensional collagen matrix models of endothelial lumen formation during vasculogenesis and angiogenesis. Methods Enzymol 443: 83-101.
